# Dr. Robert Barnwell Rhett, Jr.

**Published:** 1901-09

**Authors:** 


DR. ROBERT BARNWELL RHETT, JR., of Charleston, S. C., died at his home, August 7, 1901, aged 47 years. Dr. Rhett was a famous physician, belonged to one of the most distinguished families in South Carolina, and was a fellow of the American Association of Obstetricians and Gynecologists.

